# Serotonin synthesis, release and reuptake in terminals: a mathematical model

**DOI:** 10.1186/1742-4682-7-34

**Published:** 2010-08-19

**Authors:** Janet Best, H Frederik Nijhout, Michael Reed

**Affiliations:** 1 Department of Mathematics, The Ohio State University, Columbus, OH 43210 USA; 2 Department of Biology, Duke University, Durham, NC 27708 USA; 3 Department of Mathematics, Duke University, Durham, NC 27708 USA

## Abstract

**Background:**

Serotonin is a neurotransmitter that has been linked to a wide variety of behaviors including feeding and body-weight regulation, social hierarchies, aggression and suicidality, obsessive compulsive disorder, alcoholism, anxiety, and affective disorders. Full understanding of serotonergic systems in the central nervous system involves genomics, neurochemistry, electrophysiology, and behavior. Though associations have been found between functions at these different levels, in most cases the causal mechanisms are unknown. The scientific issues are daunting but important for human health because of the use of selective serotonin reuptake inhibitors and other pharmacological agents to treat disorders in the serotonergic signaling system.

**Methods:**

We construct a mathematical model of serotonin synthesis, release, and reuptake in a single serotonergic neuron terminal. The model includes the effects of autoreceptors, the transport of tryptophan into the terminal, and the metabolism of serotonin, as well as the dependence of release on the firing rate. The model is based on real physiology determined experimentally and is compared to experimental data.

**Results:**

We compare the variations in serotonin and dopamine synthesis due to meals and find that dopamine synthesis is insensitive to the availability of tyrosine but serotonin synthesis is sensitive to the availability of tryptophan. We conduct *in silico *experiments on the clearance of extracellular serotonin, normally and in the presence of fluoxetine, and compare to experimental data. We study the effects of various polymorphisms in the genes for the serotonin transporter and for tryptophan hydroxylase on synthesis, release, and reuptake. We find that, because of the homeostatic feedback mechanisms of the autoreceptors, the polymorphisms have smaller effects than one expects. We compute the expected steady concentrations of serotonin transporter knockout mice and compare to experimental data. Finally, we study how the properties of the the serotonin transporter and the autoreceptors give rise to the time courses of extracellular serotonin in various projection regions after a dose of fluoxetine.

**Conclusions:**

Serotonergic systems must respond robustly to important biological signals, while at the same time maintaining homeostasis in the face of normal biological fluctuations in inputs, expression levels, and firing rates. This is accomplished through the cooperative effect of many different homeostatic mechanisms including special properties of the serotonin transporters and the serotonin autoreceptors. Many difficult questions remain in order to fully understand how serotonin biochemistry affects serotonin electrophysiology and vice versa, and how both are changed in the presence of selective serotonin reuptake inhibitors. Mathematical models are useful tools for investigating some of these questions.

## Background

Traditionally, serotonin (5-HT) has been associated to a wide variety of behaviors including feeding and body-weight regulation, social hierarchies, aggression and suicidality, obsessive compulsive disorder, alcoholism, anxiety, and affective disorders[1]. In addition, 5-HT has been linked to motor system function[2], sleep-wake cycles[3], circadian rhythms[4], respiratory stability[5], embryonic development[6], and reward processing[7]. Not surprisingly, the 5-HT neurons in the nuclei originally classified by Dalhstrom and Fuxe[8] project to a large variety of regions of the central nervous system including spinal cord, cerebellum, frontal cortex, hypothalamus, hippocampus, striatum, and a bewildering variety of 5-HT receptors have been identified [9]. A huge body of research on genomics, anatomy, neurochemistry, electrophysiology, and behavior has provided a wealth of information on serotonergic systems, but the causal mechanisms of serotonergic function, both normal and in the presence of various disorders and pharmacological agents, remain largely unknown.

Polymorphisms in the serotonin reuptake transporter (SERT) gene have been associated with depression and other mood disorders[10-13] and may be associated with anxiety[14], autism[15], and suicidality[16,17]. Polymorphisms in the tryptophan hydroxylase gene have been associated with unipolar[18] and bipolar disorder[19]. Furthermore, variations in gene expression very likely play a role in the regulation of serotonergic systems both normally and in response to selective serotonin reuptake in-hibitors(SSRIs). SERTs are downregulated in the presence of SSRIs [20,21], 5-HT1A autoreceptor expression levels differ in different brain regions[22], and 5-HT1A mRNA levels are affected by gonadal hormones [23].

Because of efforts to understand the modes of action of SSRIs, the neurochemistry of serotonin has received much attention. Serotonin is synthesized in serotonergic terminals from tryptophan, which competes with tyrosine and the branched chain amino acids for transport across the blood-brain barrier[1,24]. Autoreceptors play important roles in the regulation of 5-HT chemistry. For example, 5-HT1B autoreceptors on terminals decrease synthesis and release when extracellular 5-HT rises and 5-HT1A autoreceptors affect firing rates in the dorsal raphe nucleus[25]. In addition, these regulatory mechanisms are themselves regulated by dynamic changes in autoreceptor expression levels[26]. Serotonin acts both in one-to-one neural signaling and as a neuromodulator, via volume transmission, of the effects of other neurotransmitters[1,27]. Each of these facts plays an important role in neuropsychiatry and neuropharmacology.

The electrophysiology of serotonergic signaling is related both to neurochemistry and to behavior. The classical experiments of Jacobs on cats[28] showed that the patterns of firing of nucleus centralis superior serotonergic neurons correspond to different sleep-wake states. 5-HT modulates motor firing patterns[2] and motor behavior[29,30]. Autoreceptors affect the inhibition of CA3 hippocampal pyramidal neurons caused by stimulating the ascending serotonergic pathways[31,32]. 5-HT increases the firing rates of histaminergic neurons in the hypothalamic tuberomammillary nucleus[33], inhibits the firing of somatosensory cortical neurons[34], and can inhibit or excite neurons in the ventromedial nucleus of the hypothalamus[35]. It has been proposed that 5-HT activates the hypothalamic-pituitary-adrenal axis by stimulating production of corticotropin-releasing hormone[36]. 5-HT influences dopaminergic signaling[37,38] and may affect firing in the cerebral cortex by causing the release of glutamate[39]. Traditionally, dopamine was thought to be the primary neurotransmitter involved in reward processing, but recent work suggests a strong role for 5-HT[7]. Thus, the neurochemistry and electrophysiology affect each other, both affect behavior, and both are affected, of course, by neuronal morphology, which is itself changeable.

Even this brief discussion shows why understanding the casual mechanisms in serotonergic signaling is a challenging problem. Not only does one have to understand mechanism and function on four different levels, genomic, biochemical, electrophysiological, and behavioral, but changes on each level affect function on the other three levels, and this makes the interpretation of experimental and clinical results very difficult. In addition, the brain is not fixed, but dynamical changes on different time scales are happening at all four levels. Mathematical models can play an important role because they allow one to study explicitly the simultaneous effects of all the interactions in a large complex system. Ideas and hypotheses can then be tested by *in silico *experimentation, that is, by computer simulations of the mathematical model. Our main interest is to understand how the biochemistry of 5-HT (synthesis, release, reuptake) is regulated and how the biochemistry affects the electrophysiology and vice versa. As a first step, we present in this paper a model of 5-HT biochemistry in serotonergic terminals.

**Figure 1:**
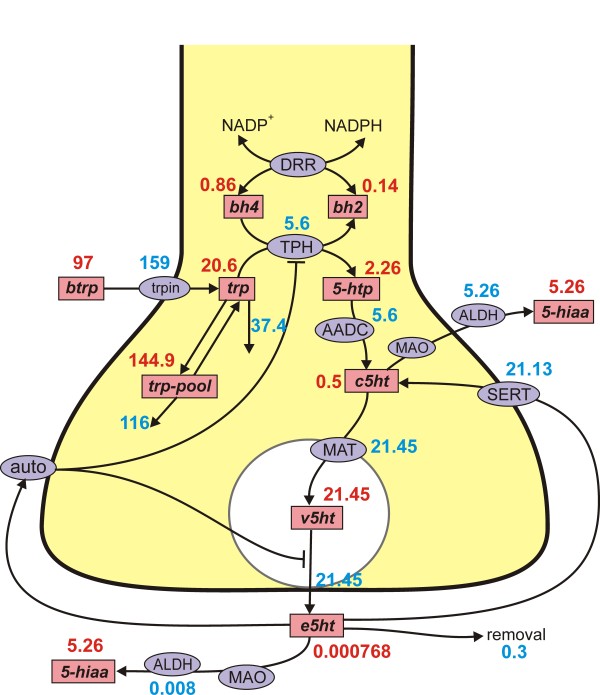
**Steady state concentrations and fluxes**. The figure shows the reactions in the model. The rectangular boxes indicate substrates and blue ellipses contain the acronyms of enzymes, transporters, and autoreceptors; steady state values in the model are indicated. Full names of the substrates are given in Table 1. Names of enzymes and transporters are as follows: Trpin, neutral amino acid transporter; DRR, dihydrobiopterin reductase; TPH, tryptophan hydroxylase; AADC, aromatic amino acid decarboxylase; MAT, vesicular monoamine transporter; SERT, 5-HT reuptake transporter; auto, 5-HT autoreceptors; MAO monoamine oxidase; ALDH, aldehyde dehydrogenase. Removal means uptake by capillaries or glial cells or diffusion out of the system.

The model includes (see Figure 1): uptake of tryptophan across the blood-brain barrier and transport into terminals; synthesis of 5-HT by tryptophan hydroxylase (THP) and aromatic amino acid decarboxylase (AADC); transport of 5-HT into a vesicular compartment by the monamine transporter (MAT); release of 5-HT into the extracellular space depending on firing rate; reuptake via the SERTs; regulation by the autoreceptors. As much as possible, the model is based on real physiology that has been determined experimentally. It is worthwhile to say at the outset that there is no such thing as "*the *serotonergic terminal"; important parameters (like SERT and autoreceptor densities) vary in different projection regions and this variation is likely to be related to function. Our main purpose is to use the model as a platform for *in silico *experimentation that sheds light on the complex regulatory mechanisms of serotonergic signaling. Some results of some simulations with the model have previously appeared elsewhere [40].

Mathematical methods have been used by a variety of authors to understand serotonergic signaling. The serotonergic model presented in this paper is conceptually similar to the dopaminergic model presented in [41]; both models were inspired by the original model of Justice *et al. *[42] for a dopaminergic terminal. Many studies use statistical methods to identify associations between variables on different levels of the serotonergic system. Cohen and colleagues used theoretical and experimental methods to show how 5-HT modulates the frequency and phase lag of bursting in lamprey spinal cord[2,43]. Butera showed by modeling how 5-HT affects the bursting behavior of neuron R15 in *Aplysia *[44]. Waggoner and colleagues introduced a three state stochastic model for the serotonin dependence of egg laying in a nematode[45]. Bunin *et al.*[46] and Daws *et al.*[47] used mathematical models and data to compute apparent values of the Michaelis-Menten constants *K*_*m*_ and *V*_*max *_for the SERTs in different projection regions. Venton *et al.*[48] used experiments and mathematical models to show that the extracellular space is well-mixed during tonic firing but not during burst firing. Kim *et al.*[4] used a mathematical model to explain why the rhythmic degradation of the mRNA of serotonin N-acetyltransferase is essential for its circadian rhythm. Tanaka *et al.*[49] used a mathematical model to show that 5-HT controls the time scale of reward prediction by differentially regulating activities in the striatum. Dayan and Huys[50] used a Markov model to study the effects of 5-HT on how the predictions of future outcomes lead to behavioral inhibition, suppression, and withdrawal and created a computational model to investigate 5-HT in affective control[51]. Stoltenberg and Nag[52] used a dynamical systems model to go directly from genes to behavior.

## Methods

The mathematical model consists of nine differential equations for the variables listed in Table 1. The differential equations corresponding to the reactions diagrammed in Figure 1 follow in Table 2. Reaction velocities or transport velocities begin with a capital V followed by the name of the enzyme, the transporter, or the process as a subscript. For example, *V*_TPH_(*trp, bh*4, *e*5*ht*) is the velocity of the tryptophan hydroxylase reaction and it depends on the concentrations of its substrates, *trp *and *bh*4, as well as *e*5*ht *(via the autoreceptors). Below we discuss in detail the more difficult modeling issues and reactions with non-standard kinetics. Table 3 gives the parameter choices and references for reactions that have Michaelis-Menten kinetics in any of the following standard forms:

**Table 1:** Names used for Variables

in equations	in text	full name
*bh*2	BH2	dihydrobiopterin
*bh*4	BH4	tetrahydrobiopterin
*trp*	Trp	tryptophan
*btrp*	serum Trp	serum tryptophan
5*htp*	5-HTP	5-hydroxytryptamine
*c*5*ht*	cytosolic 5-HT	cytosolic serotonin
*v*5*ht*	vesicular 5-HT	vesicular serotonin
*e*5*ht*	extracellular 5-HT	extracellular serotonin
5*hiaa*	5-HIAA	5-hydroxyindoleacetic acid
*trp--pool*	the tryptophan pool	the tryptophan pool

**Table 2:** The Differential Equations

(7) $$\frac{d[bh2]}{dt}=V_{\text{TPH}}\left(trp,bh4,e5ht\right)–V_{\text{DRR}}\left(bh2,\text{NADPH},bh4,\text{NADP}\right)$$
(8) $$\frac{d[bh4]}{dt}=V_{\text{DRR}}\left(bh2,\text{NADPH},bh4,\text{NADP}\right)–V_{\text{TPH}}\left(trp,bh4,e5ht\right)$$
(9) $$\frac{d[trp]}{dt}=V_{trpin}\left(btrp\right)–V_{\text{TPH}}\left(trp,bh4,e5ht\right)–V_{trp-pool}\left(trp,trp–pool\right)$$
(10) $$\frac{d[5htp]}{dt}=V_{\text{TPH}}\left(trp,bh\text{4},e\text{5}ht\right)\;–V_{\text{AADC}}\left(\text{5}htp\right)$$
(11) $$\frac{d[c5ht]}{dt}=V_{\text{AADC}}\left(5htp\right)\;–V_{\text{MAT}}\left(c5ht,v5ht\right)\;+fluox\left(t\right)V_{\text{SERT}}\left(e5ht\right)\;–V_{c5ht}^{catab}(c5ht)$$
(12) $$\frac{d[v5ht]}{dt}=V_{\text{MAT}}\left(c5ht,v5ht\right)\;–release\left(e5ht\right)\;fire\left(t\right)\;v5ht$$
(13) $$\frac{d[e5ht]}{dt}=release\left(e5ht\right)\;fire\left(t\right)\;v5ht–fluox\left(t\right)\;V_{SERT}\left(e5ht\right)\;–V_{e5ht}^{catab}(e5ht)-V_{rem}(e5ht)$$
(14) $$\frac{d[5hiaa]}{dt}=V_{c5ht}^{catab}(c5ht)+V_{e5ht}^{catab}(e5ht)-k_{hiaa}^{catab}.5hiaa$$
(15) $$\frac{d[trp-pool]}{dt}=V_{trp–pool}(trp,trp-pool)-k_{trp-pool}^{catab}\cdot trp-pool$$

**Table 3:** Kinetic Parameters (*μ*M, *μ*M/hr,/hr).

velocity	parameter	model value	literature value	references
*V*_AADC_	aromatic amino acid decarboxylase			
	*k*_*m*_	160	160	[121]
	*V*_*max*_	400		*
*V*_SERT_	serotonin transporter			
	*k*_*m*_	.17	0.05-1	[1,46,47]
	*V*_*max*_	8000		*
*V*_DRR_	dihydropteridine reductase			
	*K_bh2_*	100	4-754	[122,123]
	*K*_NADPH_	75	29-770	[124-126]
	$V_{\max}^{f}$	5000		*
	*K*_*bh*4_	10	1.1-17	[125,127]
	*K*_*NADP*_	75	29-770	[124-126]
	$V_{\max}^{b}$	3		*
*V*_MAT_	vesicular monoamine transporter			
	*K*_*m*_	.198	.123-.253	[65,66]
	*V*_*max*_	3500		*
	*k*_*out*_	40		*
*V*_TPH_	tryptophan hydroxylase			
	*K*_*trp*_	40	40	[64]
	*K*_*bh4*_	20	20	[64]
	*V*_*max*_	400		*
	*K*_*i *_(substrate inhibition)	1000	970	[64]
*V*_*trpin*_	neutral amino acid transporter			
	*K*_*m*_	64	64	[55]
	*V*_*max*_	400		*
*trp *↔	*trp*-*pool*			
	*k*_1_	6		*
	*k*_-1_	0.6		*
	catabolism and diffusion			
	$k_{trp}^{catab}$	0.2		*
	$V_{max}^{catab(c5ht)}$	1000		*
	$K_{m}^{catab(c5ht)}$	95	94-95	[81,82]
	$V_{max}^{catab(e5ht)}$	1000		*
	$K_{m}^{catab(e5ht)}$	95	94-95	[81,82]
	$k_{hiaa}^{catab}$	1	.82	[83]
	$k_{trp-pool}^{catab}$	0.2		*
	*k*_*rem*_	400		*

* see text

(1) $$V=\frac{V_{max}[S]}{K_{m}+[S]}$$

(2) $$V=\frac{V_{max}[S_{1}][S_{2}]}{\left(K_{S_{1}}+[S_{1}])(K_{S_{2}}+[S_{2}]\right)}$$

(3) V=Vmaxf[S1][S2](KS1+[S1])(KS2+[S2])−Vmaxb[P1][P2](KP1+[P1])(KP2+[P2])

for unidirectional, one substrate, unidirectional, two substrates, and bidirectional, two substrates, two products, respectively.

Table 1 gives the abbreviations used for the variables throughout. We use lower case italic abbreviations in the differential equations and other formulas so that they are easier to read. Full names for the enzymes appear in the legend to Figure 1.

### Tryptophan and the tryptophan pool

Serum tryptophan concentrations have been measured in humans and other mammals both before and after meals with different protein composition. A range of 53-85 *μ*M was found in [53] and a range of 61-173 *μ*M was found in [54]. We take as our baseline the value of 96 *μ*M found by Fernstrom in fasted rats [24]. During the experiments with our model in Results A, the serum values of tryptophan were varied corresponding to meals.

Tryptophan is transported across the blood-brain barrier by the L-transporter and is then taken up by serotonergic neuron terminals [55]. We simplify these two steps into a single step with the kinetics of the L-transporter. Choosing the right *K*_*m*_ for the L-transporter is complicated by two issues. First, the majority of tryptophan in the serum is not free but bound to albumin. Second, the other neutral and branched chain amino acids compete for the same transporter, so the effective *K*_*m *_depends on the concentrations of these other amino acids. Partridge [56] measured a *K*_*m *_= 190 *μ*M with respect to total serum tryptophan and Smith [57] measured *K*_*m *_= 15 *μ*M with respect to free serum tryptophan. We will use the effective *K*_*m *_= 330 *μ*M in the presence of other amino acids given in Kilberg [55]. We choose *V*_*max *_= 700 *μ*M/hr so that, in our model, the rate of transport into the brain (159 *μ*M/hr) closely matches that found by Kilberg (159 *μ*M/hr).

Intracellular tryptophan is used in a large number of biochemical pathways and, of course, in protein synthesis, which accounts for about half the use of tryptophan [58]. Protein breakdown and a variety of biochemical pathways are intracellular sources of tryptophan. Overall, about 2% of ingested tryptophan is used for the synthesis of serotonin [59,60]. These numbers give some crude upper and lower bounds for the percentage of intracellular tryptophan that goes to the synthesis of serotonin, but accurate estimates are not known. In dopaminergic neurons about 90% of tyrosine goes to protein synthesis and other pathways and about 10% to dopamine synthesis [61-63], so it seems reasonable to make a similar estimate for tryptophan. We let the variable *trp*-*pool *represent all the other intracellular sinks and sources of tryptophan and assume that intracellular tryptophan, *trp*, and *trp*-*pool *can be interconverted into each other:

(4) $$trp\overset{k_{1}}{\underset{k_{-1}}{↔}}trp\text{​}—\text{​}pool.$$

We choose the rate constants *k*_1 _= 6 *μ*M/hr and *k*_-1 _= .6 *μ*M/hr so that *trp*-*pool *is approximately 10 times as large as *trp*:

### Tryptophan hydroxylase

Tryptophan (*trp*) and tetrahydrobiopterin (*bh*4) are converted by tryptophan hydroxylase (TPH) into 5-hydroxytryptamine (5*htp*) and dihyro-biopterin (bh2). The velocity of the reaction, *V*_TPH_, depends on trp, *bh*4, and extracellular 5-HT (*e*5*ht*) via the autoreceptors. We take the basic kinetics from [64] with *K*_*trp *_= 40 *μ*M, *K*_*bh*4_= 20 *μ*M. TPH exhibits substrate inhibition but it is quite weak, *K*_*i *_= 1000. The second term in the velocity equation below, which represents the effect of extracellular 5-HT on synthesis rate, is discussed in detail below under "autoreceptors." The constants are chosen so that at the normal steady state (*e*5*ht *= .000768 *μ*M) this factor is equal to one, so the normal steady state is the same with and without the autoreceptors. This allows us to compare how the system changes with and without the autoreceptors when we perturb the system by changing enzyme properties, neuron firing rates, or transporter properties.

(5) $$\begin{array}{l} V_{TPH}=\frac{V_{max}(trp)(bh4)}{\left(K_{trp}+(trp)+\frac{{\left(trp\right)}^{2}}{K_{i}})(K_{bh4}+(bh4)\right)}\\ \;\;\;\;\;\;\;\;\;\cdot \left(1.5-\frac{{\left(e5ht\right)}^{2}}{\left({\left(.000768\right)}^{2}+{\left(e5ht\right)}^{2}\right)}\right) \end{array}$$

### Storage, release, and reuptake of serotonin

The 5-HTP produced by the TPH reaction is rapidly decarboxylated by the aromatic amino acid decarboxylase (AADC) to produce cytosolic serotonin. We take the parameters of AADC from the literature; see Table 3. The monoamine transporter, MAT, rapidly transports 5-HT into vesicles. We take the *K*_*m *_of the transporter to be 0.198 *μ*M as found in [65], which is consistent with the values in [66]. We choose the *V*_*max *_so that the concentration of cytosolic serotonin is very low. The experiments in [67] and the calculations in [68] in the case of dopamine suggest strongly that there is transport from the vesicles back into the cytosol, either dependent or independent of the MAT and it is likely that the same is true of serotonin [69]. We assume this transport is linear with rate constant, *k*_*out*_, chosen so that the vast majority (i.e., 98%) of the cellular serotonin is in the vesicular compartment. For simplicity we are assuming that the vesicular compartment is the same size as the non-vesicular cytosolic compartment. This assumption is unimportant since we take the cytosol to be well-mixed and we are not investigating vesicle creation, movement toward the synapic cleft, and recyling where geometry and volume considerations would be crucial. Of course, if we took the volume of the vesicular compartment to be much smaller than the volume of the cytosolic compartment, say 1 to 100, then the ratio of vesicular 5-HT concentration to cytosolic 5-HT concentration would approach the value of 10^4 ^suggested in [69].

In our model, vesicular 5-HT (*v*5*ht *in the equations) is removed from the vesicles and put into the synaptic cleft, where it becomes *e*5*ht*, by the term *release*(*e*5*ht*) *fire*(*t*) *vda*(*t*) in the differential equations for *v*5*ht *and *e*5*ht *(see the differential equations above). *fire *is a function of time in some of our *in silico *experiments, for example in Results B and C where we investigate pulse experiments and in Results E where we consider the effects SSRIs. However, for determining our baseline steady state we take *fire *= 1 *μ*M/hr, which means that vesicular serotonin is released at a constant rate such that the entire pool turns over once per hour. The term *release*(*e*5*ht*) represents the effect of *e*5*ht *on release via the autoreceptors and is discussed below. The processes by which vesicles are created, move to the synapse, and release their serotonin are complicated and interesting [67,70-72], but are not included in this model.

Extracellular serotonin has three fates. It is pumped back into the cytosol by the SERTs; it is catabolized; it is removed from the system. The *K*_*m *_= .17 *μ*M for the SERTs is taken from [46]. As we will discuss later, the *V*_*max *_will vary considerably from one projection region to another because the density of SERTs varies by at least a factor of 5. For our baseline case, we take *V*_*max *_= 4700 *μ*M/hr which is in the middle of the range, 2052-6480 *μ*M/hr, found in [46]. The function *fluox*(*t*) that multiplies the term *V*_SERT _in the differential equations for the variables *v*5*ht *and *e*5*ht *is the fraction of SERTs that remain unblocked in the presence of an SSRI. In the absence of SSRIs, *fluox*(*t*) = 1. Catabolism and removal are discussed below.

### Autoreceptors

It has been understood since the 1970s and 1980s that terminal autoreceptors (5-HT1B) sense the extracellular 5-HT concentration (*e*5*ht *in the equations). When *e*5*ht *goes up, they inhibit both the synthesis of 5-HT and the release of 5-HT from the vesicles into the synaptic cleft and when *e*5*HT *goes down they facilitate synthesis and release [9,25,73]. Thus *e*5*ht *provides a kind of end-point feedback for the entire serotonergic system from tryptophan in the serum to *e*5*HT *in the extracellular space. It is also known [74] that autoreceptors modulate reuptake, but this effect is not included in the model. Extracellular 5-HT or autoreceptor agonists can decrease synthesis by 50% [75-77] and by perhaps as much as 80-90% [78]. And, autoreceptor antagonists can increase synthesis by as much as 40-60% [77,79]. These and many other experiments are often conducted with large amounts of agonists or antagonists, which leaves open the question of what range of extracellular 5-HT causes these effects. Experiments on rats [76,80] showed that cocaine administration elevates extracellular 5-HT by factors of 2 to 5 and that such elevation has a large depressive effect on 5-HT synthesis, so it is reasonable to assume that synthesis is significantly affected by changes in *e*5*ht *over less than an order of magnitude. The second term in the formula for *V*_TPH _above contains the effect of *e*5*ht *on synthesis. When extracellular 5-HT has its steady state value of 0.768 *nM *the factor is equal to 1. As extracellular 5-HT declines towards 0, the factor increases to 1.5 and as extracellular 5-HT increases the factor declines to 0.5 (almost reaching that level when *e*5*ht *= 3 nM). Thus facilitation of synthesis can be as much as 50% and inhibition of synthesis can be as much as 50% and most of the effect is between 0-3 nM of extracellular 5-HT.

Similarly, many experiments have shown that release of vesicular serotonin can be inhibited by increased *e*5*ht *via the autoreceptors or facilitated if *e*5*ht *goes down. For example, Gothert found that release can be inhibited 65% and facilitated by 50-60% [77]. It is not certain from the experiments over what range of *e*5*ht *this effect takes place. We will assume a modest effect over a relatively small range. The factor *release*(*e*5*ht*) descends linearly from 1.5 at *e*5*ht *= 0 to 1.0 at *e*5*ht *= .000768 *μ*M, the normal steady state. Then the factor descends linearly from 1.0 at *e*5*ht *= .000768 *μ*M to 0.4 at *e*5*ht *= .0023 *μ*M. For *e*5*ht *> .0023, *release*(*e*5*ht*) remains constant at 0.4. Thus, the maximal facilitation is 50% and the maximal inhibition is 60% and the effect takes place over the range 0 - 2.3 nM of extracellular 5-HT.

### Metabolism and removal of serotonin

Serotonin is metabolized by monoamine oxidase (MAO) and aldehyde dehydrogenase (ALDH) to 5-hydroxyindoleacetic acid (5 - *hiaa*). In our simple model we are not investigating the details of catabolism, only in how *c*5*ht *and *e*5*ht *are removed from the system, so we combine these two steps into one and use the *K*_*m *_= 95 *μ*M determined in [81,82]. The rate constant for the removal of 5*hiaa *was measured to be 0.82 ± .06 in [83]; we take it to be 1/hr. This results in a model steady state concentration of *5hiaa *= 5.22 *μ*M. The ratio of 5-HIAA to 5-HT was measured to be around 1 in [84] and in the range 1-3 in [85]. Since tissue content of 5-HT in different brain regions is roughly 2-3 *μ*M [86-88], the concentration 5*hiaa *= 5.22 *μ*M is reasonable.

In our model the extracellular space is a single compartment. One should think of it as the part of the entire extracellular space corresponding to this particular synapse. Of course, if we had many model synapses, the *e*5*ht *from one will diffuse into the extracellular compartment of another (volume transmission). We are assuming for simplicity that the extracellular space is well-mixed, that is, we are ignoring diffusion gradients between different parts of the extracellular space. Venton *et al. *[48] have shown in the case of dopamine, using a combination of experiments and modeling, that the extracellular space is well-mixed during tonic firing but that substantial gradients exists between "hot spots" of release and reuptake and the rest of the extracellular space during and just after episodes of burst firing. The term *k*_*rem*_(*e*5*ht*) in the differential equation for *e*5*ht *represents removal of *e*5*ht *though uptake by glial cells, uptake by the blood, and diffusion out of the tissue. This will vary from tissue to tissue; for the base case, we chose *k*_*rem *_= 400/hr. At steady state in the base case (see Figure 1), 21.4 *μ*M/hr of *v*5*ht *is put into the extracellular space, 21.1 *μ*M/hr is put back into the cytosol by the SERTs, 0.1 *μ*M/hr is catabolized in the extracellular space, and 0.3 *μ*M/hr is removed. Thus the effect of the removal term is small at steady state but it plays a bigger role when large amounts of *v*5*ht *are dumped into the extracellular space, for example in the pulse experiments described in Results B.

**Figure 2:**
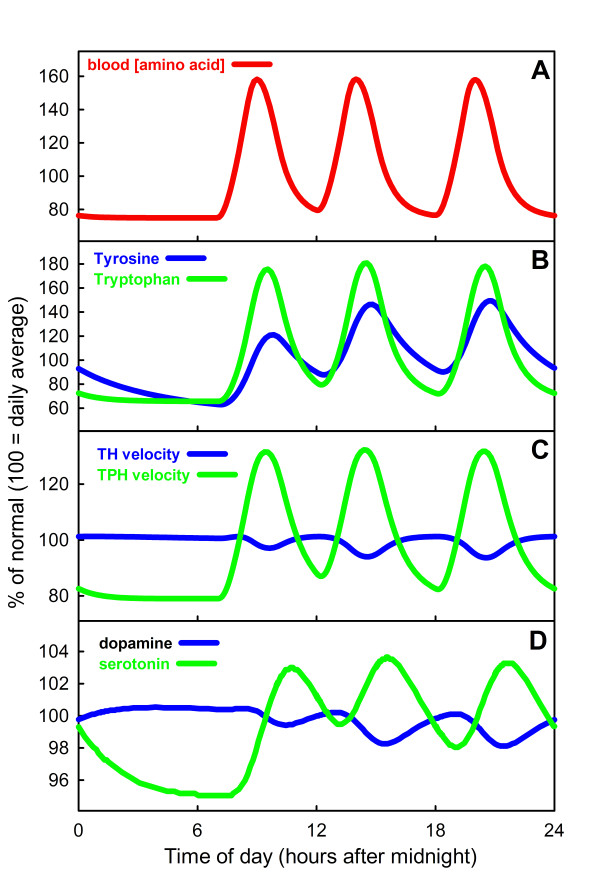
**The effect of meals on brain DA and 5-HT**. Panel A shows the blood concentration over a 24 hour period of either tyrosine or tryptophan due to three meals at 7 am, 12 pm and 6 pm. Panel B shows the tyrosine and tryptophan concentrations in dopaminergic and serotonergic synaptic terminals. Panel C shows the velocities of the TH and TPH reactions over the same 24 hour period. Panel D shows the extracellular DA and 5-HT concentrations. The vesicular stores of DA and 5-HT (not shown) vary like the extracellular concentrations in Panel D. All calculations for DA were done using the mathematical model described in [41] and the calculations for 5-HT were done using the mathematical model in this paper.

### Fluoxetine dosing

In part E of the results we use the model to investigate the results of giving a dose of the SSRI fluoxetine. The dose is represented in the model by changing the fraction of SERTs that are unblocked at any given time. The resulting function, *fluox*(*t*), multiplies *V*_SERT _in the differential equations for *c*5*ht *and *e*5*ht*. Since we give the dose at 1 hour, *fluox*(*t*) = 1 if *t *≤ 1, and for *t *≥ 1,

(6) $$fluox(t)=1-\frac{(.95){\left(t-1\right)}^{2}}{.04+{\left(t-1\right)}^{2}}\cdot e^{-(t-1)/37}.$$

The half-life of fluoxetine is quite long; 1-4 days is reported in [89]. The number 37 appearing in the exponential corresponds to a half-life of fluoxetine of a little more than a day.

### Steady state concentrations and velocities

Figure 1 shows the concentrations of all of the variables and the reaction and transport velocities at steady state.

The rate of tryptophan uptake from the serum is 159 *μ*M as found by [55]. The cellular tryptophan concentration, 20.6 *μ*M, is in the range found in most studies [86,90]. The rate of the TPH and AADC reactions, which must be equal at steady state, is in the middle of the range of values for 5-HT synthesis reported in the literature, 2.4 *μ*M/hr [91] to approximately 13 *μ*M/hr [83,92].

It is known that the cytosolic concentrations of 5-HTP and 5-HT are quite low. Fernstrom [86] measured 2 μM for 5-HTP and it is 2.26 *μ*M in the model. It is very diffcult to get reliable estimates for cytosolic 5-HT since tissue measurements include the vesicles where the concentration is known to be very high [69]. In our model cytosolic 5-HT = 0.5 *μ*M at steady state, a very low value since cytosolic 5-HT is rapidly pumped into the vesicular compartment by the monoamine transporter where the concentration is 21.45 *μ*M. The vesicular compartment turns over once an hour at steady state releasing 21.45 *μ*M/hr into the extracellular space. There, 21.13 *μ*M/hr is put back into the cytosol by the SERTs, 0.3 *μ*M/hr is removed from the system by uptake by glial cells or blood vessels or simply diffusion out of the tissue, and 0.008 *μ*M/hr is catabolized.

Most catabolism of 5-HT happens in the cytosol since the extracellular concentrations of 5-HT are so low. Of course, the cytosolic and extracellular catabolism rates plus the rate of removal must add up to the synthesis rate 5.6 *μ*M/hr. As explained above, the concentration of 5*hiaa *= 5.22 *μ*M is reasonable [84,85].

In all cases, steady states or curves showing the variables as functions of time were computed using the stiff ODE solver in MATLAB.

## Results

### A. The effect of meals on dopamine and serotonin

Since the early work of Fernstrom [93,94] it has been generally thought that dopamine synthesis is not very sensitive to tyrosine availability but that serotonin synthesis is sensitive to the availability of tryptophan [1]. We have previously constructed a model of dopamine (DA) synthesis, release, and reuptake in dopaminergic terminals [41], so we can compare and contrast the effects of meals on synthesis in dopaminergic and serotonergic terminals. The model results are displayed in Figure 2, where Panel A shows the blood amino acid concentrations, Panel B the cellular DA and 5-HT concentrations, Panel C the rates of the TH and TPH reactions, and Panel D the concentrations of DA and 5-HT in the extracellular space, during a 24 time period with three meals. In each case the y-axis indicates percent of normal. The overall conclusion is clear: there is much more variation in the 5-HT than the DA concentrations and synthesis rates. To explain why the curves look the way they do, we shall discuss each panel in turn.

It is known that blood amino acid concentrations vary dramatically depending on meal content [53-55] and also on the sequence of meals with different content [24]. During a 24 hour period the plasma amino acid concentration can vary as much as a factor of 6 but more typically varies by a factor of 2 to 4 [54]. The amino acid curves in the blood in Panel A of Figure 2 were produced by a simple model that assumes three hours of input corresponding to each meal and a relaxation time back to normal of about 6 hours after the beginning of each meal [53]. For the purpose of these model experiments we assume that the amino acid in the blood is either tyrosine or tryptophan.

Panel B shows the intracellular tyrosine and tryptophan concentrations in the dopaminergic and serotonergic terminals. These large swings in substrate availability correspond to what is seen experimentally; for example, Fernstrom found [95] that brain tyrosine can double after a meal. But why are the oscillations of tryptophan larger than the oscillations of tyrosine? In our model, the tyrosine input into the DA terminal is 241 *μ*M/hr and the tryptophan input into the 5-HT terminal is 159 *μ*M/hr corresponding to the experiments reported in [55]. However, the steady concentrations of tyrosine and tryptophan are 126 *μ*M and 21 *μ*M, respectively. Thus the tryptophan concentration is much smaller and has a much larger input and removal rate relative to its concentration than does tyrosine. This is why the percentage change due to meals is much larger in the case of tryptophan. This also explains why the tyrosine peaks increase from meal to meal, while the tryptophan peaks are all the same height because tryptophan returns almost to baseline before the next meal.

Panel C shows the velocities of the TH and TPH reactions during the 24 hour period. Despite large swings in tyrosine availability, the TH velocity remains almost constant over the 24 hour period because the reaction is running at near saturation due to the fact that the normal tyrosine concentration is well above the *K*_*m *_for tyrosine, which is 46 *μ*M in our DA model. In fact, as is clear from the graphs, the rate of the TH reaction actually goes down as tyrosine rises beyond about 100 *μ*M. This is because TH shows substrate inhibition [96]. In contrast, the oscillations in the TPH curve are large because the normal tryptophan concentration (21 *μ*M in the model) lies well below the *K*_*m *_of TPH for tryptophan (40 *μ*M in the model). Thus the rate of the TPH reaction is very sensitive to tryptophan availability. TPH also shows substrate inhibition but it is quite weak and only has an effect at very large (perhaps unphysiological) tryptophan concentrations. We have discussed the functional significance of substrate inhibition elsewhere [40,97].

Panel D shows the extracellular concentrations of DA and 5-HT over the 24 hour period. The DA concentration varies very little while the 5-HT concentration varies by about 10%. The vesicular stores of DA and 5-HT (not shown) vary similarly in the terminals; since most DA and 5-HT is in these stores, this is what one would see if one measured brain DA or brain 5-HT. Note that we assume in these model calculations that both the dopaminergic and the serotonergic neuron are firing at their tonic rates. Thus these curves give the background concentrations due to tonic firing; burst firing for short periods of time will give significant temporary deviations. Since 5-HT is thought to be an appetite suppressant [1], it makes sense that extracellular 5-HT should rise during and after meals.

### B. Release and Reuptake

**Figure 3:**
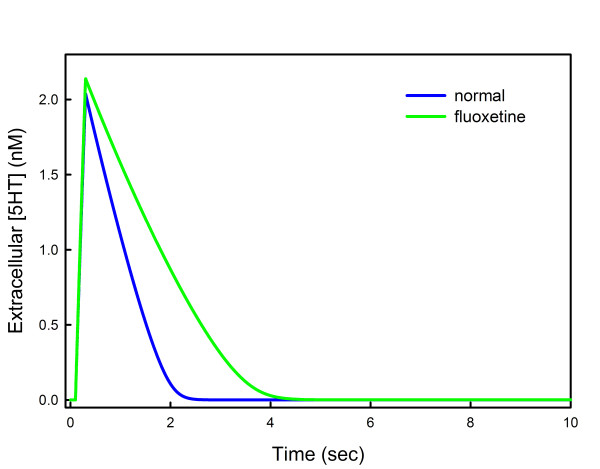
**Release and reuptake**. The time course of extracellular 5-HT is shown for a model experiment where the neuron was stimulated for 1/5 of a second (blue curve). In the presence of fluoxetine, the time course goes slightly higher and the decay time back to baseline doubles (green curve). We modeled the presence of fluoxetine by blocking half the SERTs. The curves are very similar to those in Figure six of [46].

A number of studies have examined the release of serotonin and reuptake from the extracellular space. Bunin *et al. *[46] use cyclic voltammetry and Daws *et al. *[47] use high speed chronoamperometry. Bunin *et al. *studied release and reuptake of 5-HT in rat brain slices after stimulation with electrical pulses at different frequencies. After stimulation for 1/5 of a second at 100 Hz, the extracellular 5-HT concentration in the dorsal raphe nucleus (DRN) rises to about 1.8 *μ*M and then declines rapidly back to baseline with half life of about 1 second (their Figure six). In the presence of 10 *μ*M fluoxetine, the extracellular 5-HT rises slightly higher and declines to baseline with a half-life of 2 seconds. We represent their stimulation in our model neuron by raising *fire *from 1 to 5000 for 1/5 of a second. The results can be seen in Figure 3. Extracellular 5-HT rises to 2 *μ*M and then decays back to baseline with a half-life of 1 second (blue curve). We modeled the presence of fluoxetine by blocking half the SERTs. In that case the extracellular 5-HT curve rises slightly higher and decays back to baseline with a half-life of about 2 seconds. Notice that the decay curves do not look exponential. In fact, they are linear until the concentration of extracellular 5-HT gets fairly low. This is because at 1-2 *μ*M the extracellular 5-HT concentration is well above the *K*_*m *_of the SERTs so the SERTs are saturated and pumping at a constant rate. The same effect can be seen in Figure six of [46]. The decay time back to baseline depends of course on the *V*_*max *_of the SERTs, which in turn depends on the SERT density that is quite different in different brain regions. Daws *et al. *[47] examined several different brain regions and found much longer half-lives than reported in [46], probably because those regions have much lower SERT densities. This difference may also be due to the fact that 5-HT is applied exogenously in the brains of anesthetized rats in [47] while in [46] tissue slices are stimulated by electrical pulses.

### C. SERT Knockouts

A large number of studies have examined the pharmacological and behavioral characteristics of mice that have the SERT gene knocked out. Such knockouts are of particular interest because they are an (extreme) model of what one could expect with high doses of SSRIs that block the SERTs. Table 4 shows steady state concentration and velocities for WT mice (left column) and steady state concentrations and velocities for SERT knockout mice (right column) in the model. Each column shows the steady state values if a certain fraction, *f*, of SERTs are functional. Thus, for WT mice, *f *= 1, and for SERT knockout mice *f *= 0. The intervening columns corresponds to the effects of progressively higher doses of SSRIs as one moves from left to right.

**Table 4:** Steady State Values from WT to SERT KO

	f * = 1(WT)	*f *= .5	*f *= .2	*f *= .1	*f *= .05	*f *= 0(SERT KO)
*trp*	20.1	20.9	21.1	21.1	21.2	21.3
*c*5 - *HT*	0.5	0.39	0.3	0.25	0.19	0.05
*v*5 - *HT*	21.5	19.9	18.1	17.05	14.67	6.41
*e*5 - HT **	.7	1.18	1.82	2.26	3.32	6.2
5 - *hiaa*	5.3	4.12	3.13	2.7	1.99	0.63
*V*_TPH_	5.57	4.59	3.86	3.6	3.32	3.12
*V*_MAT_	21.4	16.7	10.7	7.09	5.87	2.56
*V*_SERT_	21.1	16.2	9.93	6.16	4.5	0.0
*V*_*rem*_	0.31	0.47	0.73	0.9	1.33	2.50
*V*^*catab*^	5.26	4.12	3.13	2.7	1.99	0.63

* *f *is the fraction of SERTs that are functional.

** concentration in nM, all other concentrations in *μ*M. Velocities have units *μ*M/hr.

It is known that 5-HT tissue levels are down both in knockouts [98] and in WT mice treated with fluoxetine [99]. Homberg *et al. *[98] found that tissue levels of 5-HT drop by 50-70% and Bengel *et al. *[100] found decreases of 60-80%. In our model, vesicular 5-HT (the main determinant of tissue 5-HT) drops from 21.5 *μ*M in WT to 6.41 *μ*M in SERT knockouts corresponding well to these experimental results. Homberg *et al. *also found that 5-HIAA tissue levels decrease 45-55%; in our model, where we have a very simple model of 5-HIAA metabolism, levels decrease by 88%.

Extracellular 5-HT rises as more and more SERTs are blocked, as expected. Gainetdinov and Caron [12] report that extracellular 5-HT rises 5-6 fold in SERT knockouts and Homberg *et al. *report a 9-fold increase [98]. In our model, extracellular 5-HT rises to 6.2 *μ*M from .768 nM, a 9-fold increase. We note that, in the model, extracellular 5-HT already rises substantially when only 50% of the SERTs are active. On the other hand, vesicular 5-HT remains almost normal when only 50% of the SERTs are active, decreasing from 21.5 *μ*M to 19.9 *μ*M. This corresponds well with the finding of Bengel *et al. *[100] that SERT knockout heterozygotes had almost normal tissue levels of 5-HT.

In our model of a 5-HT terminal, we made the SERT knockout terminal by simply setting the *V*_*max *_of *V*_SERT _equal to zero. The reality is much more complicated. SERT knockout mice have been that way all their lives and one would expect that other aspects of their serotonergic systems have also changed. SERT knockouts show developmental changes in neurons and brain, an impaired hypothalamic-pituitary-adrenal axis, and desensitization of 5-HT1A and other receptors [101,102].

### D. Homeostatic effects of the autoreceptors

**Figure 4:**
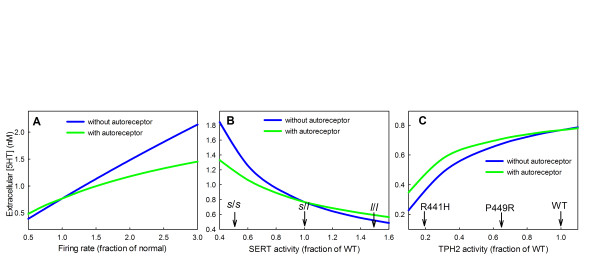
**Homeostatic effects of the autoreceptors**. Panel A shows hows extracellular 5-HT (*e*5*ht*) changes as the firing rate of the neuron varies above and below normal both with and without the autoreceptors. Panel B shows how extracellular 5-HT changes with the expression level of the SERTs both with and without the autoreceptors. s/s and l/l indicate the activities of the corresponding genotypes. Panel C shows how extracellular 5-HT changes with the activity level of TPH both with and without the autoreceptors. The activities of the R441 H and P449R polymorphims are indicated. In all cases, the autoreceptors reduce the effect of changes in firing rate and polymorphisms on extracellular 5-HT.

It has been suggested that autoreceptors provide a kind of end product inhibition that tends to stabilize extracellular 5-HT [1,103]. If extracellular 5-HT goes up, synthesis and release go down; if extracellular 5-HT goes down, synthesis and release go up. Indeed, Panel A of Figure 4 shows that extracellular 5-HT increases and decreases with the tonic firing rate, but the increase and decrease is much less in the presence of the autoreceptors. Thus the autoreceptors help to stabilize extracellular 5-HT in individuals against changes in inputs to the system like changes in firing rate or changes in mean blood tryptophan level (not shown).

However, the autoreceptors provide another kind of homeostasis, too. The genes for many of the enzymes and transporters in the serotonergic system have common polymorphisms in different human populations. Many of these polymorphisms are known to be functional in that they change the activity of the corresponding enzymes or the efficacy of the transporters. The autoreceptors tend to keep these serotonergic systems functioning normally despite the polymorphisms.

Polymorphisms in the SERT gene have been associated with depression and other mood disorders [10,11,13]. The SERT gene has a polymorphic regulatory region (the 5-HTT gene-linked polymorphic region, 5-HTTLPR), which consists of a variable tandem repeat: the short allele has 14 repeats, whereas the long allele has 16 repeats. The short allele reduces transcriptional activity of the gene and results in decreased expression of the serotonin transporter. The transcriptional activity of the short (s) allele is about 1/3 of that of the long (l) allele [14]. Although the level of transcription of a gene does not necessarily correspond to the activity of its product, we will assume that SERT activity in a s/s homozygote is 1/3 that in the l/l genotype. A study of 505 subjects [14] revealed that in a population sample of 505 individuals, 19% were of the s/s genotype, 49% were l/s, and 32% were l/l. Thus heterozygotes are the most common genotype, and if we assume their SERT activity is 1.0, then the activity of the s/s genotype would be 0.5 and of the l/l genotype 1.5. Panel B of Figure 4 shows that varying SERT activity over this range has a large effect (.5 *μ*M to 1.6 *μ*M) on the extracellular 5-HT concentration if the autoreceptors are turned off and a much smaller effect (.6 *μ*M to 1.1 *μ*M) if the autoreceptors are turned on.

There are several functional polymorphisms in the TPH2 gene and some are associated with the risk of bipolar disorder [19]. The SNP C2755A changes the amino acid from serine to tyrosine at peptide position 41; the tyrosine coding allele reduces the activity of TPH2 by about 35% [19]. A genetic polymorphism of the promoter, rs11178997, reduces TPH2 transcriptional activity by 22% [104]. The R441 H mutation of TPH2 codes for an enzyme that has only 19% of the wild type activity and the P449R mutation has an activity of 65% of wild type [105]. Thus genetic variation in human populations can cause variation of TPH2 activity between 0.19 and 1.0 of normal. Panel C of Figure 4 shows that varying TPH2 activity over this range has significant effect on the extracellular 5-HT concentration but the effect is less in the presence of the autoreceptors.

As can be seen, the autoreceptors significantly reduce the variation in extracellular serotonin caused by polymorphisms in TPH and SERT.

### E. Interaction of autoreceptors and SERTs

Many investigators have studied the effects of doses of SSRIs on extracellular 5-HT in different brain regions. A particular focus of these studies has been to understand the role of the autoreceptors. We have conducted experiments with our model that correspond to some of the experiments in [106-109].

Casanovas *et al. *[108] measured the extracellular 5-HT in the frontal cortex and the hippocampus in the rat after applying doses of 5-HT1A autoreceptor agonists. They found a rapid decline in the frontal cortex to about 30% of basal values and a decline in the hippocampus to about 70% of basal values. These effects are attributed to the stimulation of the 5-HT1A autoreceptors on cells in the raphe since it is known that such stimulation substantially decreases the firing rate of the serotonergic neurons in the raphe that project to the frontal cortex and the hippocampus. The dorsal raphe (DRN) projects to the frontal cortex and the median raphe (MRN) projects to the hippocampus. Casanovas *et al. *attribute the greater decline in the frontal cortex to the fact the density of 5-HT1A autoreceptors is higher in the DRN than the MRN [110], and therefore firing is reduced much more in the DRN than in the MRN.

**Figure 5:**
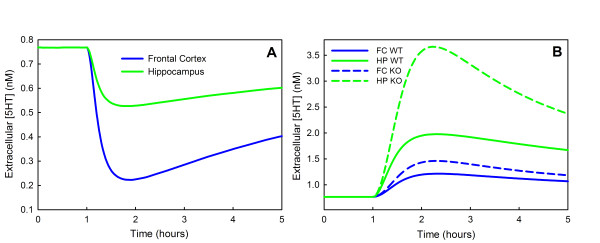
**Effects of 5-HT1A agonists and fluoxetine**. Panel A shows the change in extracellular 5-HT in the hippocampus and the frontal cortex computed by the model after a 5-HT1A agonist is given. The curves are similar to those in [108]. Panel B shows model computations of the extracellular concentrations of 5-HT in the hippocampus and the frontal cortex after a dose of an SSRI (fluoxetine or paroxetine); the solid curves are wild type and the dashed curves are 5-HT1B knockouts. These curves should be compared to Figure one (10 mg/kg dose) in [106] and Figure one (a, b, c, d) in [109]. For discussion, see the text.

In Figure 4, we see that, in the model, a reduction of *fire *to 58% of normal causes a reduction of extracellular 5-HT at steady state to 70% of normal. Therefore, to simulate the effect of a dose of a 5-HT1A agonist on the extracellular 5-HT in the hippocampus we lowered *fire *in a time-dependent manner to 58% of normal and then let it recover. Similarly, a reduction of *fire *to 20% of normal causes a reduction of extracellular 5-HT at steady state to 30% of normal. Therefore, to simulate the effect of a dose of a 5-HT1A agonist on the extracellular 5-HT in the frontal cortex we lowered *fire *in a time-dependent manner to 20% of normal and then let it recover. The effects on extracellular 5-HT in the frontal cortex and hippocampus can be seen in Panel A of Figure 5. These curves are very similar to those in Figure one (a, b) of Casanovas *et al.*[108]. The extracellular concentrations of 5-HT decrease in the frontal cortex and hippocampus because the firing rates in the DRN and the MRN are reduced due to the binding of the agonist to the autoreceptors on cell bodies. The concentrations of 5-HT in the frontal cortex and hippocampus begin to recover after the initial decline because the terminal autoreceptors in the frontal cortex and hippocampus increase synthesis and release of 5-HT.

Malagie *et al.*[106] administered fluoxetine to anaesthetized rats and measured extracellular 5-HT in the frontal cortex and hippocampus. This is a very interesting experiment because fluoxetine blocks SERTs in the DRN and MRN and thus extracellular 5-HT will rise, stimulating the 5-HT1A autoreceptors and decreasing firing as in the experiments Casanovas *et al.*[107,108]. This effect will tend to lower extracellular 5-HT in projection regions. However, fluoxetine will also block SERTs in the projection regions, which tends to raise extracellular 5-HT there. Thus, in the projection regions the level of extracellular 5-HT reflects a balance between these two effects. To see what the balance is in our model, we represent a dose of fluoxetine as described under "fluoxetine dosing" in Methods, and assume that *fire *drops as a function of time in the DRN and MRN as indicated above in the discussion of the experiments of Casanovas *et al.*. The results are shown by the blue (frontal cortex) and green (hippocampus) solid curves in Figure 5B. Hippocampal extracellular 5-HT rises by approximately 147% and frontal cortex extracellular 5-HT rises approximately 63%. These curves are very similar to the analogous curves in Malagie *et al.*, Figure one (the 10 mg/kg dose), where extracellular 5-HT rises about 110% in the hippocampus and 60% in the frontal cortex. In a second study, Malagie *et al.*[109] performed similar experiments with paroxetine on mice whose 5-HT1B autoreceptors on the terminals had been knocked out. They saw a large increase in hippocampal extracellular 5-HT and a smaller rise in frontal cortex extracellular 5-HT compared to wild type. In the model we make a 5-HT1B knockout by simply turning off the terminal autoreceptors. The results can be seen in the blue (frontal cortex) and green (hippocampal) dashed curves in Figure 5B. The model hippocampal extracellular 5-HT concentration rises 369% in the knockout, compared to 147% in wild type mice. The model frontal cortex extracellular 5-HT rises 82% as compared to 63% for the wild type. The model curves should be compared to those in Malagie *et al. *(Figure one,a,b,c,d ). They gave two doses (1 mg/kg and 5 mg/kg). Our model results are simlar to their results but with larger changes than those induced by their 1 mg/kg dose and smaller changes than than those induced by their 5 mg/kg dose. This indicates that our "dose" is between their doses. As they saw, we also found that the knockout induced smaller changes in the frontal cortex than in the hippocampus. Notice that in all cases, there is an increase in extracellular 5-HT caused by the blockage of SERTs. But this effect is then moderated by the increased rate of removal and catabolism, which slowly bring the extracellular concentrations back to equilibria (that are higher than prior to the dose of fluoxetine).

## Discussion

We have presented a relatively simple and straightforward model of synthesis, release, and reuptake of 5-HT in a serotonergic terminal. The kinetics for individual reactions and the values of constants were chosen as much as possible from the experimental literature. The purpose is to create a model that can be used, here and in future investigations, as a platform for exploring various hypotheses about serotonergic homeostasis and serotonergic signaling. Some results and predictions of the model have already appeared in [40]. We note that we have not altered parameters and kinetics to fit any particular set of experimental data. The parameter values remain the same in all the model experiments in the Results sections, except as indicated for changes corresponding to the particular experimental situations that we were examining.

Any model includes many oversimplifications. We have not included the details of the use of tryptophan in other metabolic pathways. The processes by which vesicles are created, move to the synapse, and release their serotonin are complicated and interesting [67,70-72], but are not included in this model. In our model the SERTs put released serotonin back into the terminal, but we do not include leakage of cytosolic serotonin through the SERTs into the extracellular space. We include in the model the effects of the terminal autoreceptors on serotonin synthesis (via TPH) and on serotonin release, but we do not include effects of autoreceptors on reuptake [74]. In this first model we have not included the soma explicitly, so the effects of the somatic autoreceptors are modeled by directly affecting firing rate and thus release in the terminal.

In Section A of Results we use the model to give reasons for the well known observations that dopamine synthesis is relatively insensitive to tyrosine availability, but serotonin synthesis is quite sensitive to tryptophan availability[1,93,94]. First, at the normal intracellular concentration of DA, the TH reaction is already running close to saturation, however the normal intracellular concentration of tryptophan is well below the *K*_*m *_of TPH, so changes in availability cause big changes in synthesis rate. Second, the flux into and out of the intracellular pool is much larger(relative to the pool size) in the case of tryptophan than in the case of tyrosine. We showed (Figure 2) the consequences of these differences for the time-dependent behavior of extracellular DA and 5-HT due to meals. In our model calculations, for simplicity, we assumed that the transport of the amino acids tyrosine and tryptophan across the blood brain barrier are independent of each other. In fact, both tyrosine and tryptophan compete for the L-transporter [55] with many other amino acids including the branched chain amino acids (BCAA). The protein composition of meals affects how much tyrosine and tryptophan is imported into the brain and thus how much brain DA and 5-HT change [54,95,111,112]. Even more interesting, Fernstrom[24] has shown that the order of meals affects how much tryptophan gets into the brain. The reason is that carbohydrate meals stimulate insulin production and this tends to transport amino acids into skeletal muscle, but tryptophan is partially protected from this transport because it is bound to serum albumin. A mathematical model for these competitive transport processes is in preparation.

In Section B of results we examine the time courses of extracellular 5-HT after an electric shock both with and without the presence of fluoxetine. Our time courses are very similar to those found in [46] for the DRN and substantia nigra reticulata. The shapes of the curves depend heavily on the density of SERTs, which is known to vary by a factor of 5 in different projection regions [113]. Thus, much slower uptake was found in the dentate gyrus, the corpus callosum and the CA3 region of the hippocampus [47]. This is a good reminder that there is no such thing as a single model of "*the*" serotonergic terminal. Parameters, both SERT density and also expression levels of 5-HT1A receptors [22], can vary by large amounts in different projection regions, presumably for important functional reasons.

In Section C we examined the steady state concentrations and velocities in the model corresponding to different densities of SERTs, or, equivalently, different doses of SSRIs that block the SERTs. The case where the SERTs are completely blocked corresponds to SERT knockout mice. The model concentrations of extracellular 5-HT and vesicular 5-HT are similar to those found in experiments of SERT knockout mice. Extracellular 5-HT is up 9-fold and vesicular 5-HT is down 70%. Interestingly, as more and more SERTs are blocked corresponding to higher and higher doses of fluoxetine, vesicular 5-HT decreases fairly slowly (Table 4). It is known [99] that tissue levels of 5-HT do decrease in the presence of SSRIs, but this decrease has not been remarked on very much in the literature, perhaps because it is a relatively moderate effect as predicted by our model.

In Section D we examined the steady state effects of the autoreceptors and showed that they produce two kinds of homeostasis. First, they moderate the effects of changes in the cell's environment on the concentration of extracellular 5-HT. We illustrate this by changing the firing rate (Figure 4, Panel A), but similar moderating effects are seen with changes in tryptophan availability or MAO activity. Thus, the autoreceptors allow serotonergic signaling to continue more or less as before in the face of a changed environment. Second, the autoreceptors partially compensate for the effects of various polymorphisms in the genes for TPH and SERTs (Figure 4, Panels B and C). Even though a polymorphism reduces the activity of TPH by 50%, the vesicular and extracellular 5-HT decrease by only 13% (Panel C). Thus, the autoreceptors give a kind of protection against the effects of polymorphisms. We have provided some of the first calculations that show quantitatively the importance of this aspect of autoreceptor function.

Finally, in Section E we conducted model experiments that correspond to experiments in which the time course of extracellular 5-HT was measured in different brain regions of animals after a dose of an SSRI. The purpose of many of these experiments was to investigate functional effects of the somatodendritic (5-HT1A) autoreceptors or the terminal (5-HT1B) autoreceptors, so the experiments were carried both in and without the presence of autoreceptor antagonists. In general, our model calculations correspond reasonably well to the experimental results and give some insight into the reasons why the experimental results look the way they do. We note that autoreceptor densities vary considerably in different brain regions [9,25,22], and this variation is likely to have important electrophysiological and behavioral consequences.

Other results of this model have been published previously. It is known that serotonergic neurons in the DRN fire tonically at frequencies of 0.4-2.5 spikes/second and that they also fire short bursts at higher frequencies that convey sensory or motor information [1]. In [40] we showed that the model rersponse to burst firing is very dependent on the density of SERTs on the terminal, which is proportional to the *V*_*max *_of *V*_SERT_. The size of this *V*_*max *_determines how long it takes to clear the extracellular space of excess 5-HT after a spike. If this clearance time is approximately the time between spikes during tonic ring, then even a short burst will raise extracellular 5-HT considerably. However, if this clearance time is very short, for example, 1/3 of the time between spikes in tonic firing, then a burst of three spikes at triple the tonic frequency raises extracellular 5-HT little. It is known that the density of SERTs varies by about a factor of five across different projection regions [46,47,113]. Interestingly, the frequency of tonic firing of serotonergic cells in the dorsal raphe nucleus also varies by about a factor of four or five [1,114,115]. This led us to predict that the SERT density in projection regions is tuned to the tonic firing rate of the DRN cells that project to that region, where "tuned" means that the clearance time is approximately the interspike interval for tonic firing. If it is possible to determine experimentally how the tonic firing rates of DRN cells relate to the region they project to, this prediction can be confirmed or refuted.

The fact that the mathematical model presented here is only for a serotonergic terminal limits our ability to address important issues involving mechanisms at the soma of serotonergic cells and their influence on extracellular 5-HT at terminals in projection regions. The serotonergic cells in the DRN and MRN release 5-HT from both the soma and dendrites and only 70% of the release is related to firing[25,116,117]. SSRIs block SERTs on these cell bodies as well as on terminals in projection regions, raising extracellular 5-HT in the DRN and MRN and decreasing firing rate via the 5-HT1A autoreceptors[107,118]. Thus, acute use of SSRIs can have two conflicting consequences in terminal regions: increased extracellular 5-HT because of SERT blockage on the terminal, and decreased extracellular 5-HT because of SERT blockage on the cell bodies. A common hypothesis is that chronic use of SSRIs does not have a therapeutic effect for several weeks because it takes that long for the 5-HT1A autoreceptors on cells bodies to desensitize[25,31,119]. While this hypothesis may have merit, additional factors such as SERTs are also likely to be involved. Studies have shown dramatic downregulation of SERT mRNA during chronic use of SSRIs[20,21]. It is also possible that the dynamic time scale of such SERT downregulation may contribute to the delay period. An increase in extracellular DA rapidly recruits more DATs to the terminal membrane, but downregulation of DAT activity and density follows as the increase becomes chronic[120]; similar dynamic regulation of SERTs is possible, both at the soma and at terminals. In order to examine the interplay between 5HT1A receptors and dynamic SERT regulation in the presence of SSRIs, we plan to extend our model to include the cell body of the serotonergic cell.

It is worthwhile to keep in mind how difficult the study of the serotonergic system really is. Though much new information is available that gives associations between genotypes and behaviors, the causal mechanisms are mostly unknown. These casual mechanisms necessarily involve cell biochemistry and morphology and the connections between the biochemistry and morphology and the electrophysiology of neurons and networks of neurons. Even more daunting is the fact that the four levels, gene expression, biochemical, electrophysiological, and behavioral, influence each other, both chronically and dynamically. Experiments are often difficult to interpret because changes at more than one level may be involved. In this situation, mathematical models based on real physiology can contribute to understanding, for they provide a platform for testing hypotheses and investigating how changes, chronic or dynamic, at one level cause changes at the other levels.

## Conclusions

Serotonergic systems must respond robustly to important biological signals, while at the same time maintaining homeostasis in the face of normal biological fluctuations in inputs, expression levels, and firing rates. Our mathematical model gives insight into how this homeostasis is accomplished through the cooperative effect of many different homeostatic mechanisms including the special properties of tryptophan hydroxylase, the serotonin reuptake transporters, and the serotonin autoreceptors. The model also shows how the autoreceptors moderate the effects of polymorphisms in the genes for the SERTs and TPH. The model calculations correspond quite well to a variety of experimental data. Thus, the model can be useful for testing hypotheses about the relationships between gene expression, biochemistry, and serotonergic signaling.

## Competing interests

The authors declare that they have no competing interests.

## Authors' contributions

All three authors (JB, MR, HFN) contributed equally to the formulation of the model, the estimation of parameters, experimentation with the model, the biological interpretations and conclusions, and the writing and editing of the manuscript. All authors have read and approved the final manuscript.
